# First-Line Treatment for Advanced SCLC: What Is Left Behind and Beyond Chemoimmunotherapy

**DOI:** 10.3389/fmed.2022.924853

**Published:** 2022-05-25

**Authors:** Emilio Francesco Giunta, Alfredo Addeo, Alessio Rizzo, Giuseppe Luigi Banna

**Affiliations:** ^1^Department of Medical Oncology, Candiolo Cancer Institute, FPO-IRCCS, Turin, Italy; ^2^Oncology Department, University Hospital Geneva, Geneva, Switzerland; ^3^Department of Nuclear Medicine, Candiolo Cancer Institute, FPO-IRCCS, Turin, Italy

**Keywords:** small cell lung cancer (SCLC), immunotherapy, chemotherapy, biomarkers, first-line therapy

## Abstract

Small cell lung cancer (SCLC) is still a lethal disease. Three phase III randomized clinical trials (IMpower133, CASPIAN, and KEYNOTE-604) have highlighted the survival gain of adding immune checkpoint inhibitors to first-line standard chemotherapy in advanced SCLC patients. In this review, we discuss the data from the three trials above. Furtherly, we analyze issues that still need to be elucidated, like the role of biomarkers, poor performance status at baseline, the presence of brain metastases, and the platinum compound's choice. Moreover, we depict the future of SCLC first-line therapy management, focusing on new therapeutic strategies currently under investigation.

## Introduction

Small cell lung cancer (SCLC), representing <20% of all cases of lung cancer worldwide, is still a lethal disease, with an estimated 5-year overall survival (OS) of 7% (1). The extensive stage (ES), which means the tumor is not amenable to radical radiotherapy due to its extent, is characterized by the poorest prognosis. Systemic treatments for ES disease have been implemented over the years, starting with single-agent chemotherapy (CT) in the 1970s (2). A platinum-based doublet with either etoposide or irinotecan became first-line standard CT, with a similar efficacy (i.e., median OS of ~10 months) but a different safety profile (3).

At the end of 2010s, results from three phase III randomized clinical trials, the IMpower133 (4), CASPIAN (5), and KEYNOTE-604 (6), were published. These studies have demonstrated a significant improvement in OS by adding immune checkpoint inhibitors (ICIs) to CT, thus, opening a new era in treating advanced SCLC patients.

This review will analyze some relevant aspects of the three trials above. Furtherly, we will focus on some related still open issues like potential biomarkers, poor performance status (PS), brain metastases, and the platinum compound's choice. We will then discuss the new lines of research about the first-line treatment of advanced SCLC, depicting the future in this therapeutic scenario.

## Evidence on First-Line Chemoimmunotherapy

IMpower133 is a double-blind, placebo-controlled, phase 3 trial where treatment naïve patients with ES-SCLC were randomly assigned (1:1 ratio) to receive carboplatin and etoposide with or without atezolizumab, an anti-PD-L1 antibody (4). After an induction phase consisting of four 21-day cycles, a maintenance phase with atezolizumab or placebo was offered until disease progression or unacceptable toxicity. Main patients' characteristics are resumed in Table 1. Co-primary endpoints were progression-free survival (PFS) and OS. Median PFS was 5.2 months [95% confidence interval (CI): 4.4–5.6] and 4.3 months (95% CI: 4.2–4.5) in the experimental and control arm, respectively (*p* = 0.02), while median OS was 12.3 months (95% CI: 10.8–15.9) and 10.3 months (95% CI: 9.3–11.3) in the experimental and control arm, respectively (*p* = 0.007). The objective response rate (ORR) among the two treatment groups was similar (60.2 vs. 64.4% in the experimental and control arm, respectively), as also the safety profile (4) (Table 1). The updated results with 22.9 months of median follow-up have confirmed a median OS of 12.3 and 10.3 months in the experimental and control arm, respectively (HR: 0.76, 95% CI: 0.60–0.95, *p* = 0.0154), with 34 and 21% of patients alive at 18 months in the two arms (7).

**Table 1:** Main characteristics of enrolled patients in the phase III clinical trials Impower133, CASPIAN, and KEYNOTE-604.

**Trial**	**IMpower133** **(****4****)**		**CASPIAN** **(****5****)**			**KEYNOTE-604** **(****6****)**	
	**Experimental arm**	**Control arm**	Experimental arm1^^*^^	Experimental arm2^^*^^	**Control arm**	**Experimental arm**	**Control arm**
Therapy	CbE + atezolizumab	CbE + placebo	PE + durvalumab	PE + Durvalumab + Tremelimumab	PE	PE + pembrolizumab	PE + placebo
No of patients	201	202	268	268	269	228	225
PS				NR			
−0	36.3%	33.2%	37%		33%	26.3%	24.9%
−1	63.7%	66.8%	63%		67%	73.7%	75.1%
Brain metastases at baseline	8.5%	8.9%	10%	NR	10%	14.5%	9.8%
Platinum compound				NR		
- Cisplatin	0%	0%	78%		78%	27.9% (both arms)	
- Carboplatin	100%	100%	25%		25%	68.5% (both arms)	
PFS, median (range), mo.	5.2 (4.4–5.6)	4.3 (4.2–4.5)	5.1 (4.7–6.2)	NR	5.4 (4·8–6.2)	4.5 (4.3–5.4)	4.3 (4.2–4.4)
OS, median (range), mo.	12.3 (10.8–15.9)	10.3 (9.3–11.3)	13 (11.5–14.8)	NR	10.3 (9.3–11.2)	10.8 (9.2–12.9)	9.7 (8.6–10.7)
Grade ≥ 3 AEs	58.1%	57.6%	62%	NR	62%	76.7%	74.9%

*AEs, adverse events; CbE, carboplatin + etoposide; mo, months; PE, cisplatin + etoposide; PFS, progression-free survival; NR, not reported; OS, overall survival; PS, performance status*.

* *Patients were allowed to switch between carboplatin and cisplatin at the investigator's discretion*.

CASPIAN is an open-label phase 3 trial in which untreated patients with ES-SCLC were randomly assigned (1:1:1 ratio) to receive durvalumab (anti-PD-L1 drug) plus platinum-etoposide or tremelimumab (anti-CTLA-4 antibody) and platinum-etoposide, or platinum-etoposide alone (5). Patients in the CT control arm received up to six cycles of platinum-etoposide. The immunotherapy was administered as maintenance in the experimental arms after four cycles of concomitant chemoimmunotherapy until disease progression or unacceptable toxicity. In Table 1, the main patients' characteristics are reported for the control arm and durvalumab plus platinum-etoposide arm. Median OS, the primary study endpoint, was 13.0 months (95% CI: 11.5–14.8) with durvalumab plus platinum-etoposide vs. 10.3 months (9.3–11.2) with platinum-etoposide (*p* = 0.0047). Median PFS was similar between the same two arms (5.1 vs. 5.4 months, respectively), whilst investigator-assessed ORR was higher in durvalumab than control arm (79 vs. 70%, respectively). No relevant difference in adverse events was highlighted between the two arms except for a slightly higher incidence of neutropenia and anemia in the control arm (5) (Table 1). The updated results published in 2021 substantially confirmed the OS improvement after a median follow-up time of 25.1 months, being 12.9 and 10.5 months in the experimental and control arm, respectively (HR: 0.75, 95% CI 0.62–0.91, *p* = 0.0032) (8). Notably, the addition of tremelimumab to durvalumab and platinum-based chemotherapy did not show a significant improvement in OS vs. platinum–etoposide, with a median OS of 10.4 months (95% CI: 9.6–12.0) vs. 10.5 months (9.3–11.2), respectively, but increased serious adverse events and treatment-related deaths (PMID: 33285097).

KEYNOTE-604 is a double-blind, placebo-controlled, phase 3 trial where untreated patients with ES-SCLC were randomly assigned (1:1 ratio) to receive platinum-etoposide with or without pembrolizumab, an anti-PD-1 antibody (6). The main patients' characteristics are resumed in Table 1. PFS and OS were the two primary endpoints of this study. The median PFS was 4.5 months (95% CI: 4.3–5.4) and 4.3 months (95% CI: 4.2-−4.4) in the experimental and control arm, respectively (*p* = 0.0023), while the median OS was 10.8 months (CI 95%: 9.2–12.9) and 9.7 months (95% CI: 8.6–10.7), in the experimental and control arm, respectively (*p* = 0.0164). A higher ORR was recorded in the experimental arm (70.6%) compared to the control arm (61.8%). The safety profile was similar between the two arms (Table 1).

## Potential Biomarkers

Among those biomarkers that have been explored to predict the efficacy of anti-PD-(L)1 antibodies as cancer therapy, PD-L1 is undoubtedly the most studied (9). Patients with PD-L1 positive SCLC, defined by immunohistochemical staining in over 5% of tumor cells, showed better survival in a retrospective series (10). However, another work pointed out that tumoral cells from SCLC specimens were negative for PD-L1 expression, whilst it was expressed in macrophages and correlated with tumor-infiltrating lymphocytes (TILs) (11). The different assays used to detect PD-L1 expression have made the scenario more complex (12). In the IMpower133 trial, PD-L1 testing was not performed during screening for two main reasons: an expected high rate of inadequate samples and the previous results from the phase I trial that had not shown an association between SCLC response and PD-L1 expression (4, 13). Likewise, in the CASPIAN trial, PD-L1 testing was not required for enrollment (8); it was optionally tested in archival tissue as a part of an ancillary analysis (14), confirming the low rate of PD-L1 positive tumoral cells and the lack of prognostic value when investigated as a continuous variable. In the KEYNOTE-604 trial, PD-L1 was retrospectively assessed using the combined positive score (CPS), defined as the number of PD-L1-staining cells divided by the total number of viable tumor cells times 100 (6). This estimate was based on the previous phase II KEYNOTE-158 trial (15). Patients with CPS ≥ 1%, CPS < 1% and unknown were about 40, 40, and 20%, respectively. The subgroup analyses did not observe differences between CPS ≥ 1% and CPS < 1% groups in PFS and OS. An exploratory analysis from the IMpower133 trial has not shown a predicted OS and PFS difference by each PD-L1 IHC subgroup (7).

The tumor mutational burden (TMB), an indirect measure of the tumor's neoantigen load, has been deeply investigated as a potential biomarker for immunotherapy in human cancer (16). Concerning the SCLC, data from the Checkmate 032 trial, with nivolumab vs. nivolumab plus ipilimumab in pretreated patients, suggested a role for the TMB as a potential predictive biomarker, given the high tumor responses achieved by the combination therapy in patients with high TMB compared to nivolumab (17). Similarly, the TMB did not predict either OS or PFS by an exploratory analysis of the IMpower133 trial (7). The recent FDA's approval of pembrolizumab for patients with any cancer type characterized by ≥10 mutations/megabase (mut/Mb) who had progressed to one previous treatment line without a valid alternative option has raised several criticisms. Particularly for the SCLC, it seems unlikely that clinicians will offer pembrolizumab to their patients exclusively based on a high TMB (18–20).

**Figure 1:**
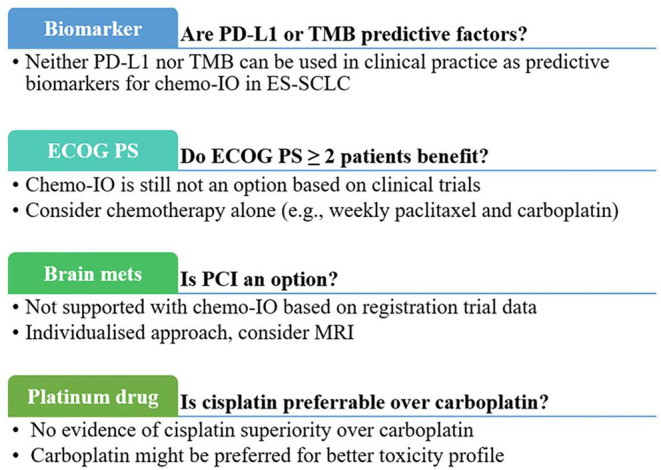
Clinical practical questions and current answers about first-line chemoimmunotherapy for extensive-stage small-cell-lung cancer. chemo-IO, chemoimmunotherapy; ECOG PS, Eastern Cooperative Oncology Group Performance Statis; mets, metastases; PCI, prophylactic cranial irradiation; PD-L1, programmed cell death ligand-1; TMB, tumor mutational burden.

In conclusion, to date, neither PD-L1 nor TMB can be used in clinical practice as predictive biomarkers for ES-SCLC (Figure 1).

## Poor Performance Status at Baseline

One of the challenging issues in treating advanced SCLC patients is their deterioration of PS before starting first-line therapy. The NCCN guidelines suggest the exclusive use of supportive care when poor PS (≥2) is not due to SCLC. In contrast, the use of systemic therapy is not discouraged when poor PS is a consequence of SCLC (21); given the high chemosensitivity of SCLC, rapid response and symptomatic improvement with CT is expected, even if at the cost of higher toxicity than patients with good PS (22, 23). However, some specific situations may require a delay in systemic treatment start, like the presence of symptomatic brain metastases or epidural/cord compression. In these cases, a priority to radiotherapy (RT) is given (21).

Chemoimmunotherapy should not be offered to ES-SCLC patients with PS ≥2 as they were not enrolled in the three mentioned phase 3 trials (4–6). A single-arm phase 2 trial is currently recruiting PS 2 patients with ES-SCLC to investigate the impact on OS of adding atezolizumab to carboplatin-etoposide, adopting the schedule of the IMpower133 trial (NCT04221529). On the other hand, there are several reports about CT alone in patients with poor PS. A single-arm phase 2 clinical trial enrolled advanced SCLC patients with PS 2 or age ≥ 70 years, showing that the combination of weekly paclitaxel (80 mg/m^2^) and carboplatin [area under the curve (AUC) 2], given on days 1, 8, 15 every 4-week cycle for up to six cycles, was feasible with few toxicities and led to a median OS of 7.2 months (24). A Japanese phase 3 randomized trial compared carboplatin plus etoposide with split doses of cisplatin plus etoposide in elderly or poor-risk SCLC patients (25). Eighteen and eight percent of enrolled patients were PS 2 and 3, respectively. Notably, PS 2-3 patients had a median OS of 8 months and PS 3 patients aged <70 years of 7 months, regardless of treatment allocation (25).

Similarly, in PS ≥ 2 non-small cell lung cancer (NSCLC) patients, the benefit of ICIs is still controversial. However, adopting frailty-assessing scales (26) or prognostic models, including the inflammatory indexes (27, 28), could assist clinical decisions. Likewise, those could be explored as helpful tools for PS2 SCLC patients (Figure 1).

## Brain Metastases in the Chemoimmunotherapy Era

Another critical aspect in the clinical management of SCLC patients is relative to their high risk of developing synchronous or metachronous brain metastases (29). Brain metastases could be symptomatic or incidental lesions at the imaging, particularly at the contrast-enhanced magnetic resonance imaging (MRI), which is more sensitive than the computed tomography scan (CT scan) (30).

Prophylactic cranial irradiation (PCI) has been offered since the 1970s to reduce the intracranial failure rate following CT in SCLC patients (31). Two randomized clinical trials demonstrated that PCI minimizes the risk of developing symptomatic brain metastases after CT, although this did not translate into a statistically significant OS benefit (32, 33). The percentage of enrolled patients who received PCI in the IMpower133 and KEYNOTE-604 was 11 and 13%, respectively, whilst in the CASPIAN trial, PCI was allowed only in the control arm after completion of CT, and 8% of patients in this arm received it (4–6). Noteworthy, in the IMpower133 trial, time to intracranial progression was longer in patients receiving CT + atezolizumab vs. CT only (20.2 vs. 10.5 months, respectively), even though they did not receive PCI (16.7 vs. 9.8 months, respectively) (34). This evidence further questioned the role of PCI in the era of chemoimmunotherapy. Furthermore, the optimal timing of PCI (before or after the CT induction phase) and the subsequent follow-up schedule remain controversial.

Therefore, in the absence of robust data supporting PCI use in patients eligible for chemoimmunotherapy, an individualized approach should be pursued considering brain magnetic resonance imaging (MRI) follow-up as a valid alternative option (35).

Moreover, brain metastases at baseline were not an exclusion criterion for the three randomized trials (4–6), provided they were asymptomatic or treated and stable off steroids and anticonvulsants. It means we do not currently have data about chemoimmunotherapy in SCLC patients with active symptomatic brain metastases, which represents a considerable proportion of diagnosed patients and remains an unmet clinical need (Figure 1).

## Chemotherapy Backbone: Cisplatin or Carboplatin

Platinum compounds are the mainstay of chemotherapeutic regimens in SCLC patients. The COCIS meta-analysis halted the long debate about the best platinum compound for ES-SCLC, showing substantial equivalence in efficacy between carboplatin and cisplatin, albeit with different safety profiles (3). Nevertheless, in the chemoimmunotherapy era, the question reappeared. In the Impower133 trial, only carboplatin was allowed (4). In the other two trials, about one-quarter of enrolled patients received cisplatin (5, 6), reflecting the clinical practice of broader adoption of carboplatin. Subgroups analyses from the two trials showed a substantial similarity between the two drugs (5, 6). Therefore, carboplatin might be favored in this setting, considering the heavier side effects of cisplatin and the need for corticosteroids as antiemetic prophylaxis (Figure 1).

## The Future of First-Line Therapy in SCLC

Several ongoing trials are evaluating the addition of an anti-PD(L)1 agent to CT in the first-line setting (Table 2). However, what is new in this setting is the investigation of other molecules in addition to chemoimmunotherapy.

**Table 2:** Ongoing clinical trials evaluating new combination strategies as first-line or maintenance therapy.

**Setting**	**Chemotherapy**	**Investigational drug(s)**	**National clinical trial number**
**CT** **+** **anti-PD-(L)1**
First-line therapy	CbE	HLX10 (anti-PD-1)	NCT04063163
First-line therapy	PE	Toripalimab (anti-PD-1)	NCT04012606
First-line therapy	Paclitaxel-albumin + Carboplatin	Shr-1210 (anti-PD-1)	NCT04790539
First-line therapy	CbE	ZKAB001 (anti-PD-L1)	NCT04878016
First-line therapy	CbE	SHR-1316 (anti-PD-L1)	NCT03711305
First-line therapy	CbE	LP002 (anti-PD-L1)	NCT04740021
**CT** **+** **anti-VEGF**
First-line therapy	PE	Anlotinib	NCT04675697
First-line therapy	PE	AL3810	NCT04254471
**CT** **+** **anti-PD-(L)1** **+** **anti-VEGF**
First-line therapy	PE	AK112 (Anti-PD-1 and VEGF Bispecific Antibody)	NCT05116007
First-line therapy	PE	Durvalumab + Anlotinib	NCT04660097
First-line therapy	PE	Toripalimab + Anlotinib	NCT04731909
First-line therapy	PE	Camrelizumab + Apatinib	NCT05001412
Maintenance therapy	No	Vorolanib + Atezolizumab	NCT04373369
Maintenance therapy	No	Camrelizumab + Apatinib	NCT04901754
Maintenance therapy	No	Tislelizumab + Anlotinib	NCT04620837
**CT** **+** **Anti-PD-1** **+** **other drugs**
First-line therapy	PE	Pembrolizumab + MK-4830 (anti-ILT4)	NCT04924101 (KEYNOTE-B99)
First-line therapy	PE	Pembrolizumab + MK-5890 (anti-CD27)	NCT04924101 (KEYNOTE-B99)
First-line therapy	PE	Sintilimab + IBI110 (anti-LAG3)	NCT05026593
First-line therapy	PE	Atezolizumab + Tiragolumab (anti-TIGIT)	NCT04256421 (SKYSCRAPER-02)
First-line therapy	PE	Durvalumab + Olaparib (PARPi)	NCT04728230
First-line therapy	PE	Tislelizumab + 177Lu-DOTATATE	NCT05142696
First-line therapy	PE	Nivolumab + BMS-986012 (fucosyl-GM1)	NCT04702880
First-line therapy	PE	Atezolizumab + LB-100 (PP2Ai)	NCT04560972
Maintenance therapy	No	Durvalumab + Ceralasertib (ATRi)	NCT04699838
Maintenance therapy	No	Atezolizumab + Lurbinectedin	NCT05091567
Maintenance therapy	No	Atezolizumab + Niraparib + Temozolomide	NCT03830918
Maintenance therapy	No	Camrelizumab + Fluzoparib (PARPi)	NCT04782089
Maintenance therapy	No	Atezolizumab + Talazoparib (PARPi)	NCT04334941
Maintenance therapy	No	Durvalumab + AZD2811 (AurKBi)	NCT04745689

*ATRi, ATR inhibitor; AurKBi, Aurora Kinase B inhibitor; CbE, carboplatin + etoposide; CT, chemotherapy; PARPi, PARP inhibitor; PE, cisplatin + etoposide; PP2Ai, Protein phosphatase 2 A inhibitor*.

The role of neoangiogenesis in SCLC is well-established, with the vascular endothelial growth factor (VEGF) and its receptor (VEGFR) as the central molecular axis involved (36–38); a higher serum concentration of VEGF correlates with poor survival (39). Bevacizumab, a humanized anti-VEGF monoclonal antibody, did not prolong the survival of advanced SCLC patients when added to CT compared to CT alone (40, 41). Antiangiogenic tyrosine kinase inhibitors (TKIs), like sorafenib and vandetanib, failed to improve the survival of chemorefractory patients (42), although they are currently under evaluation in association with CT in the first-line setting (Table 2). In the latest years, combining immunotherapy and antiangiogenic agents has been explored as a therapeutic strategy in several cancer types based on the potential synergy between these two drug classes (43); the antiangiogenic drugs could promote T-cell infiltration in tumors and reduce immunosuppression, thus enhancing the effect of immunotherapy. To date, several clinical trials have been investigating the association of chemoimmunotherapy with antiangiogenic drugs in the first-line setting and the association of ICIs and antiangiogenic agents as maintenance therapy (Table 2). Notably, the AK112, a bispecific antibody against PD-1 and VEGF, is currently being investigated with carboplatin and etoposide in a phase I trial (NCT05116007).

Other novel drugs are currently being tested with chemoimmunotherapy in the first-line setting (23). New immunomodulatory agents under investigation could potentiate the effect of anti-PD-(L)1 antibodies though their effect on specific immune targets like: the LAG3, expressed on activated T and NK cells (44); TIGIT, upregulated by activated T cells and regulatory cells (45); ILT4, expressed in myeloid cells (46); CD27, involved in T cell proliferation and differentiation to memory and effector cells (47) (Table 2). Poly ADP-ribose polymerase inhibitors (PARPi) have been approved in ovarian cancer, prostate cancer and breast cancer and are currently under investigation in SCLC, given their potential of enhancing cytotoxic response to chemotherapy, radiotherapy, and immunotherapy (48). A clinical trial with the PARPi olaparib added to chemoimmunotherapy as first-line therapy in ES-SCLC patients (NCT04728230) is ongoing. However, PARPi have currently shown limited activity in SCLC patients, suggesting that a better selection of patients is needed (49). Other drugs investigated in combination with chemoimmunotherapy are the 177Lu-DOTATATE, a somatostatin receptor-targeted radionuclide therapy; BMS-986012, an anti-fucosyl-GM1 monoclonal antibody; and LB-100, a protein phosphatase 2A (PP2A) inhibitor (Table 2).

In parallel, translational research focused on identifying specific subgroups of patients who do benefit—or do not—from immunotherapy. In the latest years, immune signatures have been developed and studied in several cancer types (50). Specifically for SCLC, two recently published works shed light on this topic. Xie et al. have built up a prognostic 10-gene immune-related signature (ARAF, HDGF, INHBE, LRSAM1, NR1D2, NR3C1, PLXNA1, PML, SP1, and TANK), able to predict SCLC patients' survival; however, this model needs validation as a predictive tool for immunotherapy (51). Gay et al. have identified four SCLC subtypes based on the expression of three transcription factors (i.e., ASCL1, NEUROD1, and POU2F3); if those are all not expressed, an inflamed gene signature showed a similar correlation between SCLC subtypes and their vulnerability to specific drugs (52). Also for this molecular classification, validation is needed mandatory.

## Conclusions

The addition of ICIs to standard chemotherapy represents a milestone in the first-line therapeutic scenario of ES-SCLC. Results from the three phase III randomized clinical trials are consistent, with OS gain across all patients' subgroups. However, primary resistance to chemoimmunotherapy is still challenging for ES-SCLC patients. More research efforts are needed to answer specific questions, like identifying responding patient according to their clinical and molecular characteristics, adding novel anticancer drugs to chemoimmunotherapy, and optimizing the therapeutic strategy for patients with symptomatic brain metastases.

## Author Contributions

AA and GLB: conceptualization and supervision. EG: writing—original draft and methodology. AR and GLB: validation and review and editing. All authors contributed to the article and approved the submitted version.

## Funding

GLB's work was supported by FPRC 5xmille Ministero Salute 2017 PTCRC-Intra 2020 CTU-Lung; Italian Ministry of Health, Ricerca Corrente 2022.

## Conflict of Interest

GLB reports personal fees from Janssen Cilag, Boehringer Ingelheim, and Roche. AA reports personal fees from BMS, Astrazeneca, Roche, Pfizer, MSD, Boehringer. The remaining authors declare that the research was conducted in the absence of any commercial or financial relationships that could be construed as a potential conflict of interest.

## Publisher's Note

All claims expressed in this article are solely those of the authors and do not necessarily represent those of their affiliated organizations, or those of the publisher, the editors and the reviewers. Any product that may be evaluated in this article, or claim that may be made by its manufacturer, is not guaranteed or endorsed by the publisher.

## References

[1] Cancer.Net Editorial Board. Lung Cancer - Small Cell: Statistics. (2022). Available online at: https://www.cancer.net/cancer-types/lung-cancer-small-cell/statistics (accessed March 21, 2022).

[2] Karim SM Zekri J. Chemotherapy for small cell lung cancer: a comprehensive review. Oncol Rev. (2012) 6:e4. 10.4081/oncol.2012.e4 25992206 PMC4419639

[3] Rossi A Di Maio M Chiodini P Rudd RM Okamoto H Skarlos DV. Carboplatin- or cisplatin-based chemotherapy in first-line treatment of small-cell lung cancer: the COCIS meta-analysis of individual patient data. J Clin Oncol. (2012) 30:1692–8. 10.1200/JCO.2011.40.4905 22473169

[4] Horn L Mansfield AS Szczesna A Havel L Krzakowski M Hochmair MJ. First-line atezolizumab plus chemotherapy in extensive-stage small-cell lung cancer. New Engl J Med. (2018) 379:2220–9. 10.1056/NEJMoa1809064 30280641

[5] Paz-Ares L Dvorkin M Chen Y Reinmuth N Hotta K Trukhin D. Durvalumab plus platinum-etoposide versus platinum-etoposide in first-line treatment of extensive-stage small-cell lung cancer (CASPIAN): a randomised, controlled, open-label, phase 3 trial. Lancet. (2019) 394:1929–39. 10.1016/S0140-6736(19)32222-6 31590988

[6] Rudin CM Awad MM Navarro A Gottfried M Peters S Csoszi T. Pembrolizumab or placebo plus etoposide and platinum as first-line therapy for extensive-stage small-cell lung cancer: randomized, double-blind, Phase III KEYNOTE-604 study. J Clin Oncol. (2020) 38:2369–79. 10.1200/JCO.20.00793 32468956 PMC7474472

[7] Liu SV Reck M Mansfield AS Mok T Scherpereel A Reinmuth N. Updated overall survival and PD-L1 subgroup analysis of patients with extensive-stage small-cell lung cancer treated with atezolizumab, carboplatin, and etoposide (IMpower133). J Clin Oncol. (2021) 39:619–30. 10.1200/JCO.20.01055 33439693 PMC8078320

[8] Goldman JW Dvorkin M Chen Y Reinmuth N Hotta K Trukhin D. Durvalumab, with or without tremelimumab, plus platinum-etoposide versus platinum-etoposide alone in first-line treatment of extensive-stage small-cell lung cancer (CASPIAN): updated results from a randomised, controlled, open-label, phase 3 trial. Lancet Oncol. (2021) 22:51–65. 10.1016/S1470-2045(20)30539-8 33285097

[9] Davis AA Patel VG. The role of PD-L1 expression as a predictive biomarker: an analysis of all US Food and Drug Administration (FDA) approvals of immune checkpoint inhibitors. J Immunother Cancer. (2019) 7:278. 10.1186/s40425-019-0768-9 31655605 PMC6815032

[10] Ishii H Azuma K Kawahara A Yamada K Imamura Y Tokito T. Significance of programmed cell death-ligand 1 expression and its association with survival in patients with small cell lung cancer. J Thorac Oncol. (2015) 10:426–30. 10.1097/JTO.0000000000000414 25384063

[11] Schultheis AM Scheel AH Ozretic L George J Thomas RK Hagemann T. PD-L1 expression in small cell neuroendocrine carcinomas. Eur J Cancer. (2015) 51:421–6. 10.1016/j.ejca.2014.12.006 25582496

[12] Udall M Rizzo M Kenny J Doherty J Dahm S Robbins P. PD-L1 diagnostic tests: a systematic literature review of scoring algorithms and test-validation metrics. Diagn Pathol. (2018) 13:12. 10.1186/s13000-018-0689-9 29426340 PMC5807740

[13] Chiang AC Sequist LVD Gilbert J Conkling P Thompson D Marcoux JP. Clinical activity and safety of atezolizumab in a phase 1 study of patients with relapsed/refractory small-cell lung cancer. Clin Lung Cancer. (2020) 21:455–63.e454. 10.1016/j.cllc.2020.05.008 32586767

[14] Goldman JW Garassino MC Chen Y Ozguroglu M Dvorkin M Trukhin D. Patient-reported outcomes with first-line durvalumab plus platinum-etoposide versus platinum-etoposide in extensive-stage small-cell lung cancer (CASPIAN): a randomized, controlled, open-label, phase III study. Lung Cancer. (2020) 149:46–52. 10.1016/j.lungcan.2020.09.003 32961445

[15] Chung HC Piha-Paul SA Lopez-Martin J Schellens JHM Kao S Miller WHJr. Pembrolizumab after two or more lines of previous therapy in patients with recurrent or metastatic SCLC: results from the KEYNOTE-028 and KEYNOTE-158 studies. J Thorac Oncol. (2020) 15:618–27. 10.1016/j.jtho.2019.12.109 31870883

[16] Sha D Jin Z Budczies J Kluck K Stenzinger A Sinicrope FA. Tumor mutational burden as a predictive biomarker in solid tumors. Cancer Discov. (2020) 10:1808–25. 10.1158/2159-8290.CD-20-0522 33139244 PMC7710563

[17] Hellmann MD Callahan MK Awad MM Calvo E Ascierto PA Atmaca A. Tumor mutational burden and efficacy of nivolumab monotherapy and in combination with ipilimumab in small-cell lung cancer. Cancer Cell. (2018) 33:853–61.e854. 10.1016/j.ccell.2018.04.001 29731394 PMC6750707

[18] Addeo A Banna GL Weiss GJ. Tumor mutation burden-from hopes to doubts. JAMA Oncol. (2019) 5:934–5. 10.1001/jamaoncol.2019.0626 31145420

[19] Prasad V Addeo A. The FDA approval of pembrolizumab for patients with TMB >10 mut/Mb: was it a wise decision? No. Ann Oncol. (2020) 31:1112–4. 10.1016/j.annonc.2020.07.001 32771305

[20] Addeo A Friedlaender A Banna GL Weiss GJ. TMB or not TMB as a biomarker: that is the question. Crit Rev Oncol Hematol. (2021) 163:103374. 10.1016/j.critrevonc.2021.103374 34087341

[21] Ganti AKP Loo BW Bassetti M Blakely C Chiang A D'Amico TA. Small cell lung cancer, version 2.2022, NCCN clinical practice guidelines in oncology. J Natl Compreh Cancer Network. (2021) 19:1441–64. 10.6004/jnccn.2021.0058 34902832 PMC10203822

[22] Demedts IK Vermaelen KY van Meerbeeck JP. Treatment of extensive-stage small cell lung carcinoma: current status and future prospects. Eur Respir J. (2010) 35:202–15. 10.1183/09031936.00105009 20044461

[23] Cortinovis D Bidoli P Canova S Colonese F Gemelli M Lavitrano ML. Novel cytotoxic chemotherapies in small cell lung carcinoma. Cancers. (2021) 13:152. 10.3390/cancers13051152 33800236 PMC7962524

[24] Neubauer M Schwartz J Caracandas J Conkling P Ilegbodu D Tuttle T. Results of a phase II study of weekly paclitaxel plus carboplatin in patients with extensive small-cell lung cancer with Eastern Cooperative Oncology Group Performance Status of 2, or age > or = 70 years. J Clin Oncol. (2004) 22:1872–7. 10.1200/JCO.2004.11.023 15143079

[25] Okamoto H Watanabe K Kunikane H Yokoyama A Kudoh S Asakawa T. Randomised phase III trial of carboplatin plus etoposide vs split doses of cisplatin plus etoposide in elderly or poor-risk patients with extensive disease small-cell lung cancer: JCOG 9702. Br J Cancer. (2007) 97:162–9. 10.1038/sj.bjc.6603810 17579629 PMC2360311

[26] Friedlaender A Banna GL Buffoni L Addeo A. Poor-performance status assessment of patients with non-small cell lung cancer remains vague and blurred in the immunotherapy era. Curr Oncol Rep. (2019) 21:107. 10.1007/s11912-019-0852-9 31768759

[27] Banna GL Cortellini A Cortinovis DL Tiseo M Aerts J Barbieri F. The lung immuno-oncology prognostic score (LIPS-3): a prognostic classification of patients receiving first-line pembrolizumab for PD-L1 >/= 50% advanced non-small-cell lung cancer. ESMO Open. (2021) 6:100078. 10.1016/j.esmoop.2021.100078 33735802 PMC7988288

[28] Banna GL Tiseo M Cortinovis DL Facchinetti F Aerts J Baldessari C. Host immune-inflammatory markers to unravel the heterogeneous outcome and assessment of patients with PD-L1 >/=50% metastatic non-small cell lung cancer and poor performance status receiving first-line immunotherapy. Thorac Cancer. (2022) 13:483–8. 10.1111/1759-7714.14256 34939342 PMC8807213

[29] Lukas RV Gondi V Kamson DO Kumthekar P Salgia R. State-of-the-art considerations in small cell lung cancer brain metastases. Oncotarget. (2017) 8:71223–33. 10.18632/oncotarget.19333 29050358 PMC5642633

[30] Seute T Leffers P ten Velde GP Twijnstra A. Detection of brain metastases from small cell lung cancer: consequences of changing imaging techniques (CT versus MRI). Cancer. (2008) 112:1827–34. 10.1002/cncr.23361 18311784

[31] Yu NY Sio TT Ernani V Savvides P Schild SE. Role of prophylactic cranial irradiation in extensive-stage small cell lung cancer. J Natl Compr Canc Netw. (2021) 19:1465–9. 10.6004/jnccn.2021.7105 34902829

[32] Slotman B Faivre-Finn C Kramer G Rankin E Snee M Hatton M. Prophylactic cranial irradiation in extensive small-cell lung cancer. N Engl J Med. (2007) 357:664–72. 10.1056/NEJMoa071780 17699816

[33] Takahashi T Yamanaka T Seto T Harada H Nokihara H Saka H. Prophylactic cranial irradiation versus observation in patients with extensive-disease small-cell lung cancer: a multicentre, randomised, open-label, phase 3 trial. Lancet Oncol. (2017) 18:663–71. 10.1016/S1470-2045(17)30230-9 28343976

[34] Higgins KA Curran WJ Jr Liu SV Yu W Brockman M Johnson A. Patterns of disease progression after carboplatin/etoposide + atezolizumab in extensive-stage small-cell lung cancer (ES-SCLC). Int J Radiat Oncol Biol Phys. (2020) 108:1398. 10.1016/j.ijrobp.2020.09.020 33427656

[35] Picardi C Caparroti F Di Maio M Kassak F Banna GL Addeo A. Prophylactic cranial irradiation in extensive disease small cell lung cancer: an endless debate. Crit Rev Oncol Hematol. (2019) 143:95–101. 10.1016/j.critrevonc.2019.08.010 31563079

[36] Lucchi M Mussi A Fontanini G Faviana P Ribechini A Angeletti CA. Small cell lung carcinoma (SCLC): the angiogenic phenomenon. Eur J Cardiothorac Surg. (2002) 21:1105–10. 10.1016/S1010-7940(02)00112-4 12048093

[37] Tanno S Ohsaki Y Nakanishi K Toyoshima E Kikuchi K. Human small cell lung cancer cells express functional VEGF receptors, VEGFR-2 and VEGFR-3. Lung Cancer. (2004) 46:11–9. 10.1016/j.lungcan.2004.03.006 15364128

[38] Tas F Duranyildiz D Oguz H Camlica H Yasasever V Topuz E. Serum vascular endothelial growth factor (VEGF) and interleukin-8 (IL-8) levels in small cell lung cancer. Cancer Invest. (2006) 24:492–6. 10.1080/07357900600814771 16939957

[39] Salven P Ruotsalainen T Mattson K Joensuu H. High pre-treatment serum level of vascular endothelial growth factor (VEGF) is associated with poor outcome in small-cell lung cancer. Int J Cancer. (1998) 79:144–6. 10.1002/(sici)1097-0215(19980417)79:2<144::aid-ijc8>3.0.co;2-t 9583728

[40] Ready NE Dudek AZ Pang HH Hodgson LD Graziano SL Green MR. Cisplatin, irinotecan, and bevacizumab for untreated extensive-stage small-cell lung cancer: CALGB 30306, a phase II study. J Clin Oncol. (2011) 29:4436–41. 10.1200/JCO.2011.35.6923 21969504 PMC3221525

[41] Spigel DR Townley PM Waterhouse DM Fang L Adiguzel I Huang JE. Randomized phase II study of bevacizumab in combination with chemotherapy in previously untreated extensive-stage small-cell lung cancer: results from the SALUTE trial. J Clin Oncol. (2011) 29:2215–22. 10.1200/JCO.2010.29.3423 21502556

[42] Schneider BJ Kalemkerian GP. Personalized therapy of small cell lung cancer. Adv Exp Med Biol. (2016) 890:149–74. 10.1007/978-3-319-24932-2_9 26703804

[43] Song Y Fu Y Xie Q Zhu B Wang J Zhang B. Anti-angiogenic agents in combination with immune checkpoint inhibitors: a promising strategy for cancer treatment. Front Immunol. (2020) 11:1956. 10.3389/fimmu.2020.01956 32983126 PMC7477085

[44] Goldberg MV Drake CG. LAG-3 in cancer immunotherapy. Curr Top Microbiol Immunol. (2011) 344:269–78. 10.1007/82_2010_114 21086108 PMC4696019

[45] Chauvin JM Zarour HM. TIGIT in cancer immunotherapy. J Immunother Cancer. (2020) 8:57. 10.1136/jitc-2020-000957 32900861 PMC7477968

[46] Gao A Sun Y Peng G. ILT4 functions as a potential checkpoint molecule for tumor immunotherapy. Biochim Biophys Acta Rev Cancer. (2018) 1869:278–85. 10.1016/j.bbcan.2018.04.001 29649510

[47] Starzer AM Berghoff AS. New emerging targets in cancer immunotherapy: CD27 (TNFRSF7). ESMO Open. (2020) 4 (Suppl. 3):e000629. 10.1136/esmoopen-2019-000629 32152062 PMC7082637

[48] Barayan R Ran X Lok BH. PARP inhibitors for small cell lung cancer and their potential for integration into current treatment approaches. J Thorac Dis. (2020) 12:6240–52. 10.21037/jtd.2020.03.89 33209463 PMC7656434

[49] Knelson EH Patel SA Sands JM. PARP inhibitors in small-cell lung cancer: rational combinations to improve responses. Cancers. (2021) 13:727. 10.3390/cancers13040727 33578789 PMC7916546

[50] Thorsson V Gibbs DL Brown SD Wolf D Bortone DS Ou Yang TH. The immune landscape of cancer. Immunity. (2018) 48:812–30.e814. 10.1016/j.immuni.2018.03.023 29628290 PMC5982584

[51] Xie Q Chu H Yi J Yu H Gu T Guan Y. Identification of a prognostic immune-related signature for small cell lung cancer. Cancer Med. (2021) 10:9115–28. 10.1002/cam4.4402 34741430 PMC8683526

[52] Gay CM Stewart CA Park EM Diao L Groves SM Heeke S. Patterns of transcription factor programs and immune pathway activation define four major subtypes of SCLC with distinct therapeutic vulnerabilities. Cancer Cell. (2021) 39:346–60.e347. 10.1016/j.ccell.2020.12.014 33482121 PMC8143037

